# Highlight: 40th Anniversary Virtual Issues: Epigenetics and Evolution

**DOI:** 10.1093/gbe/evae187

**Published:** 2024-09-04

**Authors:** Casey L McGrath

To celebrate the 40th anniversary of the first issue of *Molecular Biology and Evolution*, the Society for Molecular Biology and Evolution (SMBE) journals are publishing a series of virtual issues in 2024. These virtual issues contain selected papers published in the society's journals, *Molecular Biology and Evolution* and *Genome Biology and Evolution*. Each virtual issue is accompanied by a Perspective that highlights the historic and contemporary contributions of our journals to a specific topic in molecular evolution.

September's Perspective, titled “Epigenetics Research in Evolutionary Biology: Perspectives on Timescales and Mechanisms” appears in *Molecular Biology and Evolution* and was written by Soojin Yi. The accompanying virtual issues—one for *Molecular Biology and Evolution* and one for *Genome Biology and Evolution*—include noteworthy papers on Epigenetics and Evolution published in the two journals and can be found at SMBE's 40th anniversary site.

